# Route towards efficient magnetization reversal driven by voltage control of magnetic anisotropy

**DOI:** 10.1038/s41598-021-88408-z

**Published:** 2021-04-22

**Authors:** Roxana-Alina One, Hélène Béa, Sever Mican, Marius Joldos, Pedro Brandão Veiga, Bernard Dieny, Liliana D. Buda-Prejbeanu, Coriolan Tiusan

**Affiliations:** 1 Faculty of Physics, Babes-Bolyai University, Cluj-Napoca, Cluj, Romania; 2 Univ. Grenoble Alpes, CEA, CNRS, G-INP, IRIG-SPINTEC, Grenoble, France; 3 Technical University of Cluj-Napoca, Cluj-Napoca, Romania

**Keywords:** Magnetic properties and materials, Electronic devices, Magnetic properties and materials

## Abstract

The voltage controlled magnetic anisotropy (VCMA) becomes a subject of major interest for spintronics due to its promising potential outcome: fast magnetization manipulation in magnetoresistive random access memories with enhanced storage density and very low power consumption. Using a macrospin approach, we carried out a thorough analysis of the role of the VCMA on the magnetization dynamics of nanostructures with out-of-plane magnetic anisotropy. Diagrams of the magnetization switching have been computed depending on the material and experiment parameters (surface anisotropy, Gilbert damping, duration/amplitude of electric and magnetic field pulses) thus allowing predictive sets of parameters for optimum switching experiments. Two characteristic times of the trajectory of the magnetization were analyzed analytically and numerically setting a lower limit for the duration of the pulses. An interesting switching regime has been identified where the precessional reversal of magnetization does not depend on the voltage pulse duration. This represents a promising path for the magnetization control by VCMA with enhanced versatility.

## Introduction

In an era dominated by an increasing demand of information processing speed and efficient information storage, spintronics can offer novel solutions to circumvent some of the limitations presently reached by conventional microelectronics technologies^1–3^. In the last decade, magnetization manipulation by electric fields entered the race for energy efficient methods for information storage when the very first mentions of electric field manipulation of magnetization in ferromagnetic semiconductors and 3*d* metals opened this research direction^4,5^. It was shown experimentally that by using an electric field in order to control the magnetization dynamics, it is possible to achieve writing with only a few fJ/bit^5–7^. A wide series of theoretical and experimental studies have been dedicated to this subject, covering the topics of material optimization for enhanced voltage control of magnetic anisotropy (VCMA) as well as magnetization dynamics control^8,9^.

The idea of voltage control of magnetization relies on the phenomenon of magnetic anisotropy modulation with an electric field. This effect can be due to several mechanisms: (i) charge depletion and accumulation at the ferromagnet(FM)/oxide(Ox) interface, in the case of purely electronic effects^10^ and/or (ii) oxygen migration in the case of ionic mechanisms^11,12^. Another very likely source of electric-field control of the magnetization is the Rashba spin–orbit mechanism, where the underlayer in contact with the FM layer is a heavy metal (HM)^13^. In some cases, the HM/FM interface can have a greater contribution to the VCMA effect than the FM/Ox interface^13^. Many first principle studies have been dedicated to this topic, generally for systems composed of a heavy metal, a 3*d* ferromagnet and a dielectric, typically MgO^14–17^. Simultaneously, experimental evidence of voltage controlled magnetic anisotropy (VCMA) has emerged in a wide range of structures, and static and dynamic measurements have been performed in order to explore its limits^18^. However, from the micromagnetic and macrospin simulations point of view, this field is not explored enough and a deep understanding of the behavior of magnetization in this framework and of its governing parameters is still needed. Some studies on VCMA using the micromagnetic framework aim at explaining the magnetization reversal process under the influence of an electric field, with some design approaches being proposed^19–21^. A broad variety of macrospin studies exist, oriented towards the optimization of system operation through pulse shape^22,23^, thermal stability^24^ and towards decreasing of the write-error-rate (WER), some of which accompanied by experimental studies^18,24–26^. Other macrospin studies focus on the association between VCMA and spin transfer torque (STT) methods of magnetization manipulation^27^ or on the VCMA and a Rashba field which is used as an in-plane bias magnetic field^25^. A detailed fundamental study of the dynamics is dedicated to a design where an assisting magnetic field is absent^28^ and the switching relies purely on the fine tuning of the voltage pulse duration. However, a reliable fast switching process is conditioned by extremely short pulses of precise length. Therefore, we considered it appropriate to search for a less restrictive regime from an application point of view.

In this work, we carried out a thorough study of the precessional switching in the VCMA framework assisted by an external magnetic field, including the calculation of switching diagrams in the macrospin approximation, as such a detailed approach is still missing. We discuss the behavior of magnetization in response to simultaneously applied stimuli (voltage modulation of anisotropy and a magnetic field applied in plane). Therefore, we will not refer to a specific system, nor to a specific saturation magnetization value, anisotropy constant or field, but we will employ values that are close to those which are generally obtained in experimental systems and usually observed in simulations. The modelled system and the parameters used are described in the following sections. We will show that there is a recipe for reaching a regime where the switching probability is no longer sensitive to the electric field pulse duration, which is quite important from an application point of view. Also, we show that in the VCMA framework, a change of paradigm concerning the damping coefficient takes place: for spin transfer torque (STT) a small value is preferred, but in VCMA, this is no longer the case.

## Results

For the macrospin simulations, we considered a ferromagnetic disk of thickness *t* = 0.9 nm, saturation magnetization *M*_*s*_ = 1000 kA/m and an out-of-plane effective anisotropy field of µ_0_*H*_*K*_ = 80 mT. These parameters are rather usual values for ferromagnetic thin films exhibiting perpendicular magnetic anisotropy (PMA)^29,30^ and particularly, they are typical for heavy-metal/ferromagnet/oxide systems most common in VCMA studies^14,21,31,32^. This model satisfies magnetic entities with lateral dimensions bellow the domain wall width, which in this case is around 72 nm.

**Figure 1:**
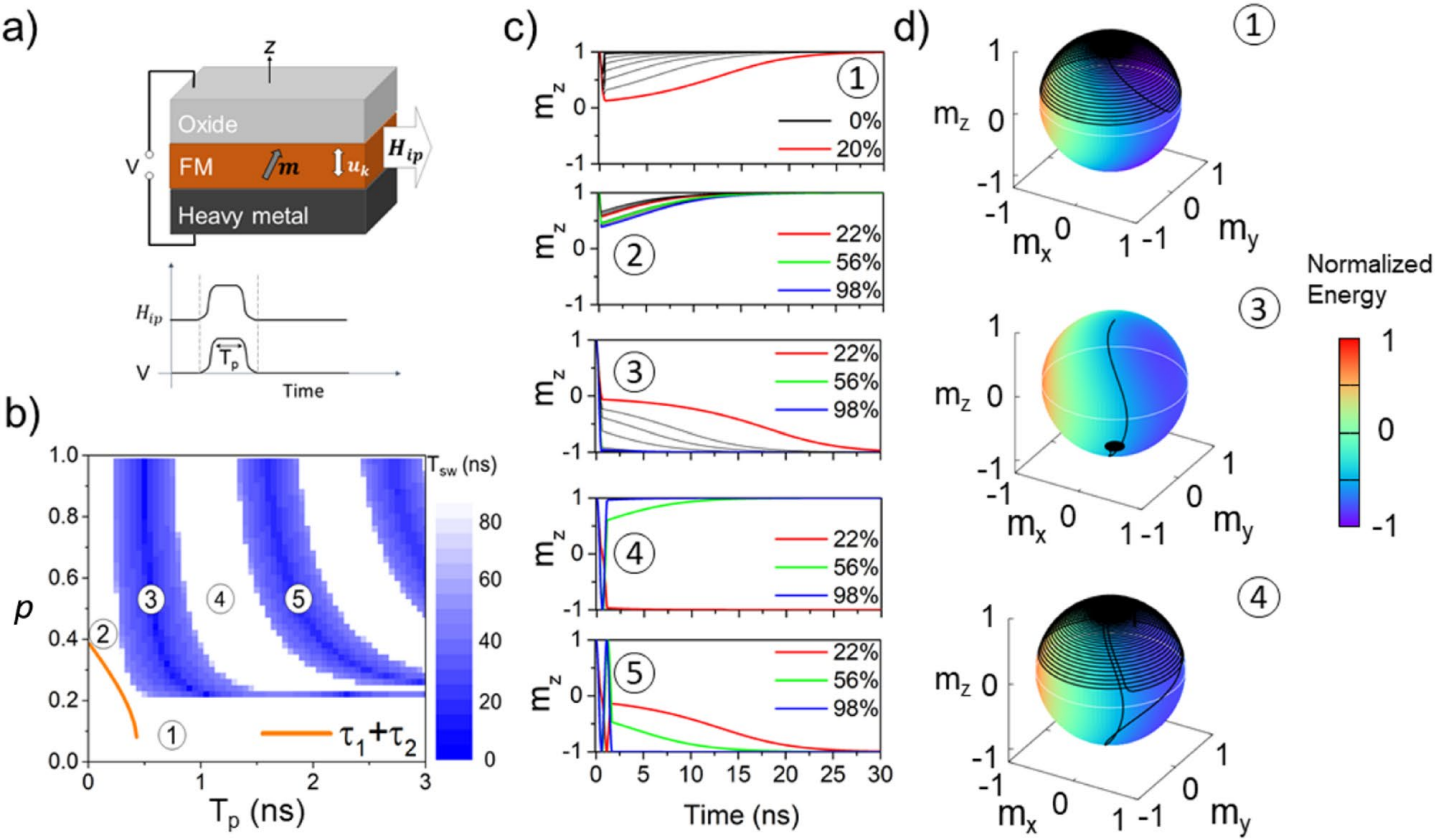
(**a**) Schematic of the system, where *u*_k_ represents the anisotropy field direction, ***m*** is the normalized magnetization vector and *T*_p_ is the voltage/magnetic field pulse duration; (**b)** Switching diagram for *H*_*ip*_ = 0.0324 T, α = 0.01 where the switching probability is correlated with the switching time, *T*_sw._. The orange line represents an analytical estimation of the minimum pulse length (sum of critical times τ_1_ and τ_2_) that allows the crossing of the *xOy* plane; (**c**) Dynamic behavior of the *Oz* projection of the magnetization vector, *m*_z_, in different regions of the diagram; (**d**) Associated magnetization trajectories (black lines) on the normalized energy sphere for successful and unsuccessful switching events; *m*_x_, *m*_y_ and *m*_z_ represent the *Ox*, *Oy* and *Oz* projections of the normalized magnetization vector; the color code represents the normalized total free energy of the system under applied electric and magnetic fields pulses.

At rest, the magnetization lies spontaneously along the *Oz* axis, parallel to the anisotropy field, *H*_*K*_. In order to trigger the magnetization switching driven by VCMA, an electric field is applied to reduce the energy barrier between the + *Oz* and  − *Oz* magnetization states, as well as a dynamic stimulus, such as a magnetic field applied in-plane, *H*_*ip*_, exerting a torque on the magnetization, responsible of a precessional motion^31^. In our simulations, we considered that the electric field is applied through a voltage pulse synchronized with an in-plane magnetic field pulse, applied independently with respect to the voltage pulse. The fact that the in-plane field H_ip_ is applied simultaneously with the voltage pulse prevents additional initial precession around the effective anisotropy axis (issued from competition between the PMA and in-plane H_ip_) and lead to a final magnetization damped relaxation, after the precessional switch, towards -m_z_ and not along the H_ip_-PMA effective quantization axis. This is schematically shown in Fig. 1a. The in-plane field *H*_*ip*_ may have several origins, ranging from an applied DC field, exchange-bias or Rashba effect^25^, (a more detailed discussion of this aspect is available in the supplementary section). Such a field can be synchronized with the voltage pulse in such a manner that it would restore the effective field along the *Oz* direction after the pulse is cut, thus eliminating the concerns regarding a decrease of the thermal stability factor.

The response of the system excited by synchronous voltage and magnetic field pulses is summarized in the diagram shown in Fig. 1b for varying $p$ and $T_{p}$, where *p* represents the modulation of the anisotropy field ***H***_***K0***_, expressed as a percentage proportion of the anisotropy field *H*_*K*_ = (1 − p)*H*_*K0*_. The amplitude of the magnetic field is µ_0_*H*_*ip*_ = 32mT (~ 40% of *H*_*K0*_) while the duration of the pulse is varied at the nanosecond scale. The color code of the diagram Fig. 1b is associated with the switching time defined as the time at which the magnetization reaches the reversed state after the application of the pulse. Numerically a limit of 90 ns is set (white color) indicating that the magnetization has not switched. We can observe that the switching diagram displays alternating reversal and non-reversal domains above a certain critical modulation, as well as a narrow horizontal band, where the switching probability is rendered independent of the pulse duration. Five regions are identified, and their dynamic characteristics are presented in Fig. 1c and d. Overall, the precession takes place around an effective field ***H***_***eff***_, which is the sum of ***H***_***K***_ and ***H***_***ip***_ and is controlled by the effective field orientation towards the *Oz* axis, as well as by the pulse duration.

In region 1, the magnetic anisotropy modulation is too small, and switching is not possible, regardless of pulse length. On the normalized energy surface, the minimum energy in the upper hemisphere is closer to the *Oz* axis, around ***H***_***eff***_, indicating a small reduction of the energy barrier. This behavior can be observed until the precession cone is tangent to the *xOy* plane, i.e. until a critical modulation of the anisotropy is reached. In region 2, although the switching does not occur, the reason behind this behavior is different. The magnetization requires a certain amount of time to reach the film plane, and this depends on ***H***_***eff***_. However, no matter how we modulate the anisotropy, if the pulse length is not long enough, the precession is transferred too fast from ***H***_***eff***_ back to ***H***_***K***_. The first blue stripe where the switching occurs for the first time is denoted in the diagram by region 3. This region contains a resonant switching regime, where an accurate pulse duration control is crucial for maximizing the switching probability. Figure 1d also reveals that in order to achieve a fast-resonant switching, it is not necessary to completely suppress the anisotropy barrier between the + *Oz* and  *− Oz* states. Related to this region, an analytical estimation of the minimum pulse length for which the magnetization crosses the *xOy* plane and relaxes in the  *− Oz* space is graphically represented by the thick orange line. This pulse duration is expressed as the sum of critical times τ_1_ and τ_2_ whose detailed expressions and significance are given in the Discussion section and does not account for damping. Region 4 depicts a situation where the pulse duration is long enough in order to allow the magnetization to relax on the *-Oz* direction and to go back to the initial state crossing the *x*O*y* plane twice. This behavior is limited in terms of pulse duration, longer pulses leading to a regime where the switching is again possible. This can be observed in region 5, where three successive reversals between + *Oz* to  *− Oz* end up with the magnetization along  *− Oz* direction. The diagram follows a pattern of resonant switching, alongside its superior harmonics, where the fastest switching takes place in region 1, for a pulse duration equal to a half of its precession period. Concerning the switching probability, it is clear that odd multiples of the half-precession period will favor the switching, while even multiples will place the magnetization back along the + *Oz* axis.

**Figure 2:**
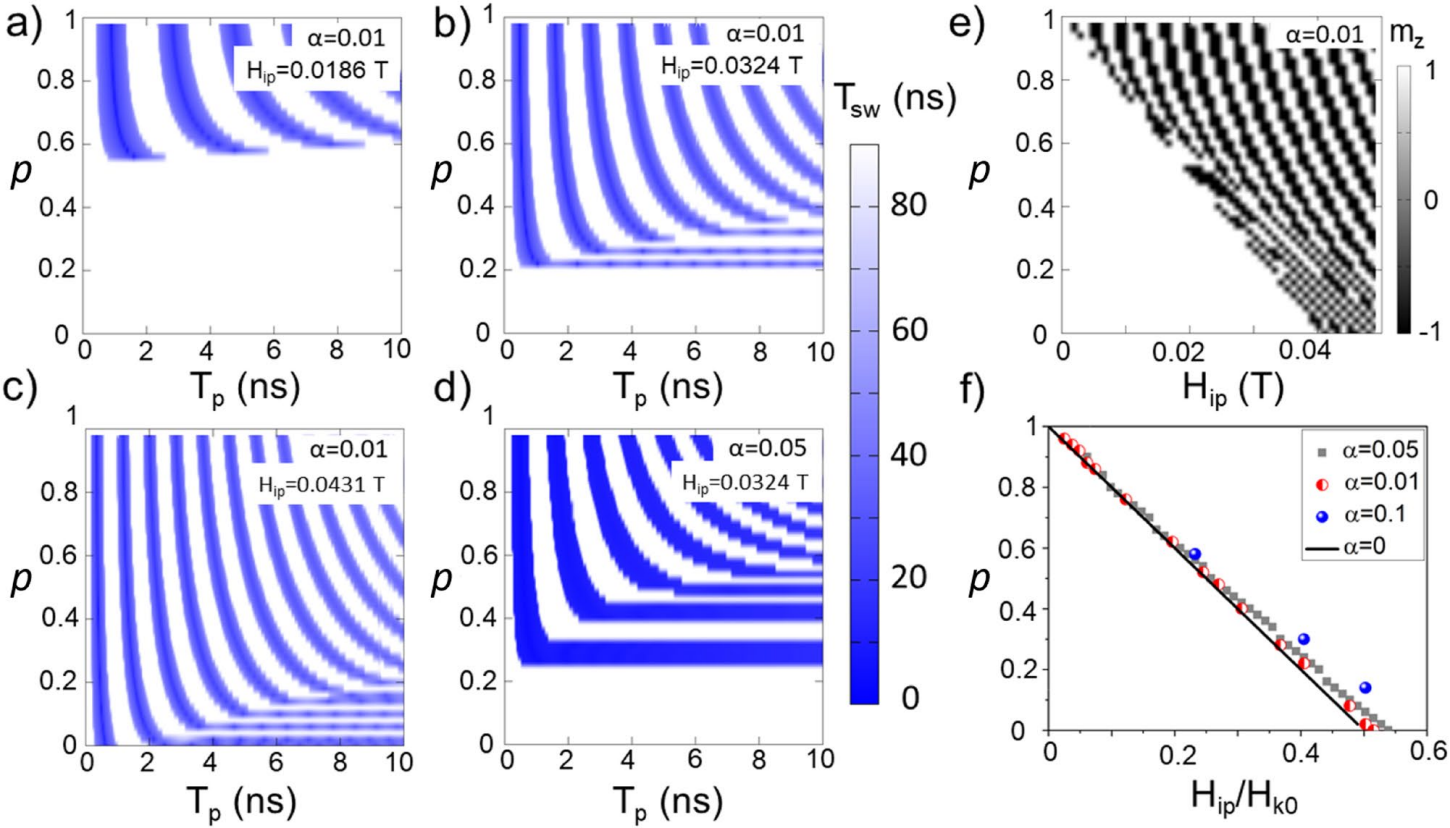
Switching diagrams (**a**–**c**) were obtained for the same damping value α = 0.01; (**d**) switching diagram for α = 0.05 for the same *H*_*ip*_ value as in (**b**). (**e**) Switching diagram for a pulse duration of 10 ns, with α = 0.01, where a switching probability of “1” is associated to a *m*_z_ magnetization projection value of “ − 1”, corresponding to the  *− Oz* axis orientation. (**f**) Critical modulation of anisotropy for which switching processes independent on the pulse duration are possible for various damping values (α = 0 is taken from Eq. (3)).

The position and width of the regular bands are affected both by the in-plane field amplitude and the damping parameter, as one can see on the diagrams from Fig. 2a–d. The diagrams display a critical modulation value to initiate the resonant switching (region 3) except the one from Fig. 2c for which the amplitude of the in-plane magnetic field is more than 50% of the anisotropy field. So far in literature, the diagrams reported are similar to those in Fig. 2c since the ratio between *H*_*ip*_ and *H*_*K*_ is large^18,27,33^. Figure 2d shows similarities with Fig. 2b, but a higher damping value (α = 0.05) broadens the switching bands and appears to reduce the switching time. A shift in the critical band towards larger modulation coefficient values *p* is remarked. The different representation of a switching diagram at fixed *T*_*p*_, *p* and *H*_*ip*_, represented in Fig. 2e clearly shows that if the pulse duration is equal to 10 ns for each *H*_*ip*_ there is a critical modulation coefficient *p* below which there are no switching events.

By systematically varying the in-plane magnetic field amplitude as well as the damping parameter value, we have extracted the critical modulation *p*_c_ which appears to decrease linearly with *H*_*ip*_/*H*_*k0*_ and vanishes if *H*_*ip*_ becomes larger than half of the amplitude of the anisotropy field (Fig. 2f).

## Discussion

### Critical modulation

**Figure 3:**
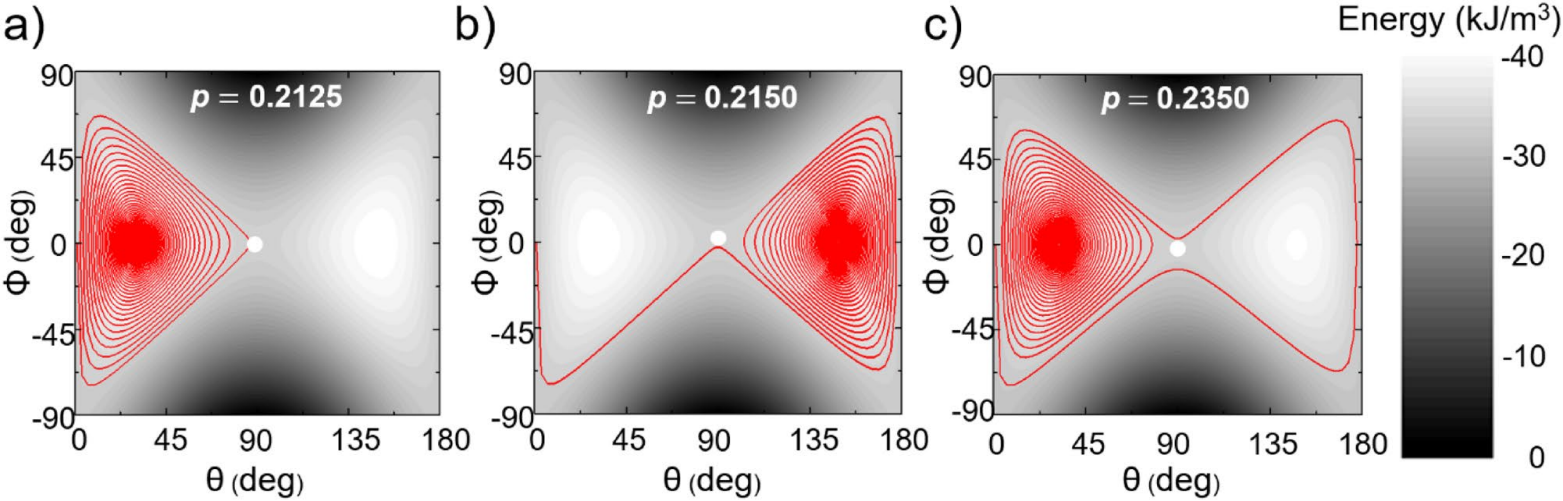
Magnetization dynamics for below critical (*p* = 0.2125), at critical (*p* = *p*_c_ = 0.2150) and above critical MCA modulation (*p* = 0.2350), for µ_0_*H*_*ip*_ = 0.0324 T and α = 0.01. The trajectories are described in terms of standard polar ϕ and θ angles. The pulse duration is *T*_*p*_ = 10 ns.

To get a deeper understanding of the linear dependence of the critical modulation on the in-plane magnetic field, one must analyze the trajectories described by the magnetization on the energy landscape in polar coordinates. Figure 3 shows the magnetization trajectories for three cases corresponding to a modulation below, equal to and above the critical value. If the modulation is too small (*p* = 0.2125), the magnetization approaches the *xOy* plane, but it cannot touch the plane (*m*_z_ ≠ 0), being pulled back to the effective field and finally relaxing towards + *Oz* once the pulses are stopped. If the modulation is equal to the critical value *p* = *p*_*c*_ = 0.2150, the magnetization reaches a saddle-point with *m*_*x*_ = 1 and *m*_*z*_ = 0, which is the equivalent of the hard-plane orientation, then it successfully passes this point and relaxes towards the *-Oz* axis. This regime is maintained until *p* reaches the value of 0.2350, when the magnetization does not relax in the *z* < 0 space, but a second crossing of the hard plane occurs, so that the magnetization switches back to its initial state. One can notice the magnetization ringing to reach an equilibrium state, this feature being controlled both by the damping parameter and the MCA modulation coefficient, *p*.

Based on the relation describing the free energy of the system (see Methods), it is straightforward to see that the energy surface has a saddle-point corresponding to the in-plane hard axis ($\theta = 90^\circ ,\phi = 0^\circ$) and two minima which are symmetric with respect to the *x*-axis as shown in Fig. 3.

The primary criterion to commute between the two minima is that the magnetization trajectory passes through the saddle point^34^, meaning that the energy of the initial state should be at least equal to that of the saddle point. The three cases presented in Fig. 3 supports this bifurcation criterion. In our simulations, the magnetization is initially perfectly aligned with the *z*-axis and thus the critical modulation $p_{c}$ should satisfy the relation:

1 $$- \frac{1}{2}\mu_{0} \left( {1 - p_{c} } \right) H_{K0} { }M_{s} = - \mu_{0} M_{s} H_{ip}$$

in the limit of negligible damping. This simple model demonstrates that the critical modulation decreases linearly with the in-plane applied magnetic field:

2 $$p_{c} { } = 1 - \frac{{H_{ip} }}{{H_{K0} /2}}$$

Moreover, the critical modulation exists only if the *H*_*ip*_ value stays below a half of the anisotropy field *H*_*K0*_. The continuous line from Fig. 2f agrees very well with the numerical results for decreasing damping values.

### Switching time

**Figure 4:**
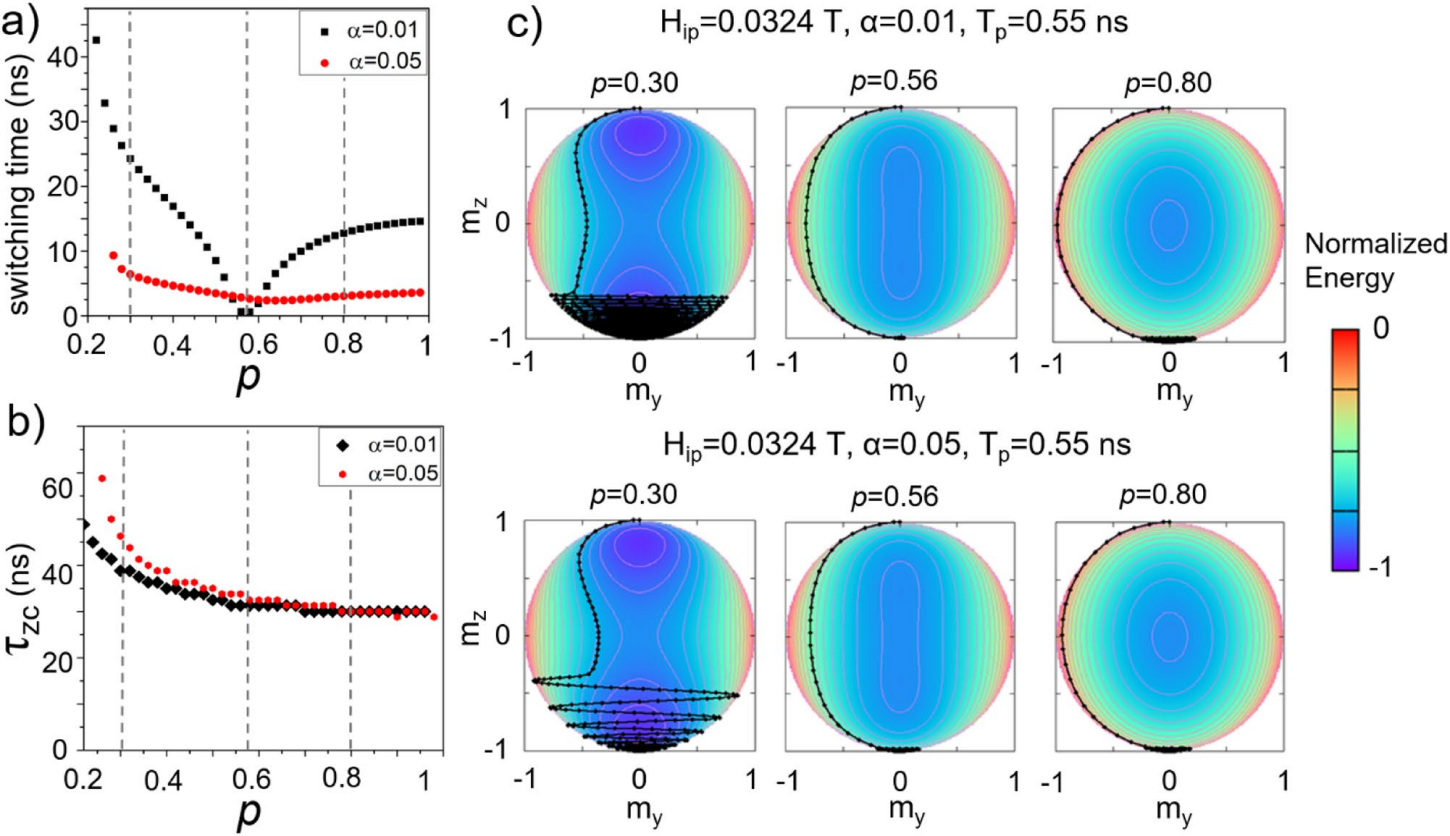
(**a**) Switching time corresponding to the first resonant switching stripe for two different values of the damping constant (pulse duration of 0.55 ns, *H*_*ip*_ = 0.0324 T). (**b**) Evolution of the zero-crossing time *τ*_*zc*_ necessary for *m*_*z*_ to reach the xOy plane versus the anisotropy modulation *p* for α values of 0.01 and 0.05. (**c**) The magnetization trajectories corresponding to *p* values of 0.30, 0.56 and 0.80 (dashed lines in (**a**) and (**b**)) are represented on a normalized energy surface for *α* values of 0.01 (top row) and 0.05 (bottom row).

One parameter valuable from applicative point of view is the switching time, the time that the magnetization takes to commute between the two stable states + /− Oz. The dependence of the switching time on the MCA modulation corresponding to the first stripe of switching from Fig. 1b, is not a monotonous function (Fig. 4a). For a small damping value α = 0.01, an optimal range of MCA modulation is identified around *p* = 0.56 for which the pulse duration (T_p_ = 0.55 ns) is precisely equal to the lapse of time taken by the magnetization to go from + Oz to  − Oz. It is thus possible to achieve a sub-nanosecond switching (resonant switching) but within a very narrow range of MCA modulation. Otherwise, if the pulse has not this precise value (or the MCA modulation is not at the optimal value), the magnetization rings for a long time to get to the equilibrium since the energy dissipation rate, proportional with the damping is very small. This drawback due to the ringing is obvious if we consider the zero-crossing time—τ_zc_ dependence on the MCA modulation (Fig. 4b). Once the pulses are applied, the magnetization relaxes very quickly towards the hard plane (below 0.5 ns). This zero-crossing time, defined as m_z_(τ_zc_) = 0, appears to be slightly dependent on the damping value and to decrease when the MCA modulation increases. For most of the MCA values, the zero-crossing time τ_zc_, is short with respect to the overall switching time except at the optimal condition where the switching time is roughly twice the τ_zc_. The ringing of the magnetization (see trajectories from Fig. 4c) pushes the switching time above 10 ns. However, one can notice that a larger value of the damping constant (α = 0.05) leads in average to a decrease of the switching time since the ringing of the magnetization is reduced by the larger rate of energy dissipation. Reasonably short switching time (below 5 ns) can be achieved for a moderate voltage modulation of the anisotropy. While for the spin-transfer torque (STT) driven magnetization switching, a small damping value is more suitable in terms of current densities and energy consumption^35^, here in the VCMA approach, a larger value of the damping constant is preferred.

From applicative point of view, tailoring the pulse duration in an accurate manner with 100 ps resolution is difficult. Thus, we find it more appealing to explore systems where this would cease to be a rigid constraint, even if this might lead to longer switching time than the resonant switching. Tailoring the switching time by damping constant seems to be a possible path, as indicated by the dynamics represented on energy surfaces in Fig. 4. In Fig. 4a, one can identify a second advantage: for the same anisotropy modulation, except for a very restricted *T*_p_ range, a faster switching is achieved for α = 0.05 than α = 0.01, which means that less energy consumption is needed for the process to take place. This beneficial effect is obvious only when looking at the total switching time which is relevant for applications. If we look at the time necessary for *m*_*z*_ to cross the hard-plane (*m*_*z*_ = 0), we see that the trend seems to be completely opposite, as the damping pulls the magnetization harder towards the potential minimum, making it more difficult to reach close to the saddle point. This reveals the important ringing attenuation provided by *α*.

This cut-through the region 1 of Fig. 1b (pulse duration of 0.55 ns, 50% modulation of anisotropy) shows that upon increasing the damping value, the magnetization reversal approaches a resonant switching mechanism. The shortest switching time is obtained when the pulse duration is adjusted so that the precession follows a ballistic pole-to-pole trajectory. This corresponds to a pulse duration equal to a half of a precession period, for anisotropy modulation coefficient larger than the critical value *p*_c_. Using the LLG equation (see Eq. (7) Methods) dominated by the precession term, an estimation of the resonant switching time is possible, with a good agreement with the numerical results in the ideal case of full anisotropy modulation (*p* = 1):

3 $$\tau_{rs} \cong \frac{{\pi \left( {1 + \alpha^{2} } \right)}}{{\gamma \mu_{0} H_{ip} }}$$

**Figure 5:**
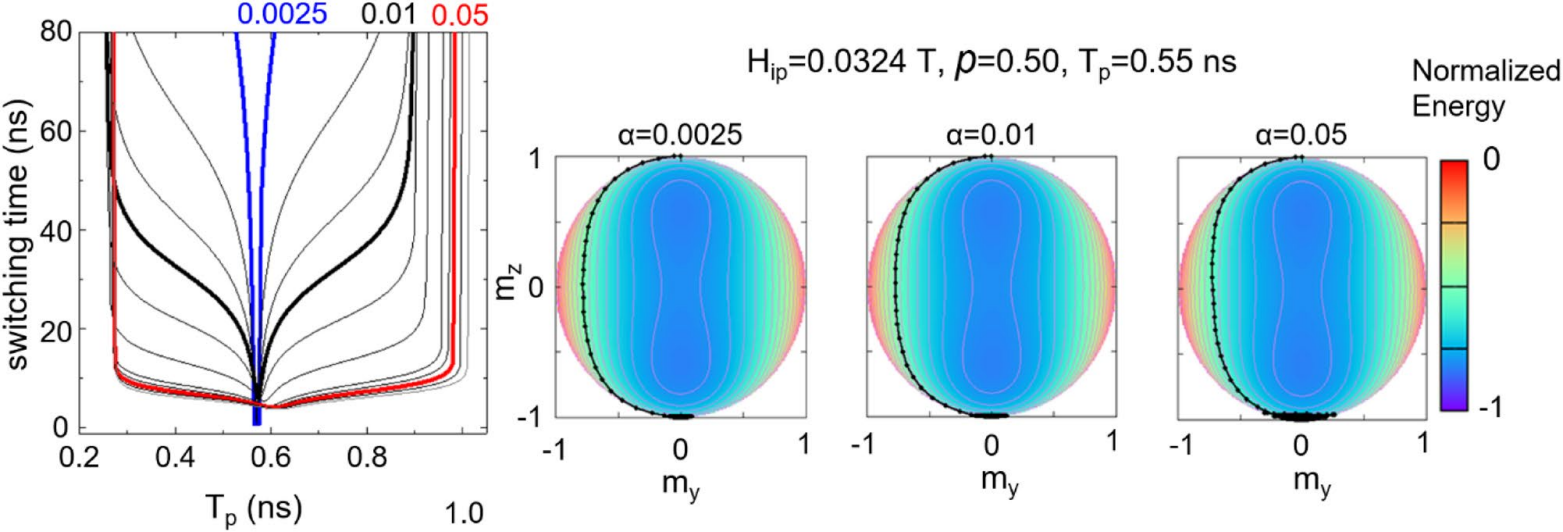
Slice through the first resonant switching line, for *α* = 0.0025, 0.01 and 0.05, *H*_*ip*_ = 0.0324 T and 50% modulation in anisotropy. Corresponding magnetization trajectories for different α values are represented on a normalized energy surface.

The optimal condition to have the fastest magnetization switching corresponds to a pulse duration such as $T_{p} = \tau_{rs}$. This criterion predicts a shift of the minimum switching time in relation with the pulse duration depending on the damping value as confirmed by the Fig. 5. It is worth mentioning that as the value of the damping constant α increases, one can obtain similar switching times for a pulse duration close to $\tau_{rs}$, thus a very fine tuning of the pulse duration is not mandatory anymore. This emphasizes the aforementioned observation that, for an application, the damping pathway in VCMA is a promising direction for obtaining reliable magnetization switching. The dynamics displayed on the energy surfaces for *p* = 0.50 for different damping values reveal that an increased damping pulls the trajectory closer to the saddle point.

The idea of a larger damping value that broadens the possibility of magnetization reversal independent of pulse duration is most visible in the critical modulation bands. As we mentioned before, a larger damping will result in a wider critical band (Fig. 2d, horizontal blue bands of region 3,5), but we did not yet explain the conditions in which such bands appear in the switching diagrams. In essence, in this region the magnetization reversal probability is 100%, because no matter how long the applied pulses are, if the duration is longer than half of the precession period, the magnetization will lose energy and relax along the  *− Oz* direction. This region property might be very useful in terms of switching reliability. Although it was shown that large damping values tend to increase the thermal fluctuations effect on the dynamics and thus the write-error-rate^25^, Matsumoto et al.^36^ showed that there is a minimum in the write-error-rate even at large damping values, following a stripe pattern that resembles the switching probability diagram (Fig. 2d). Therefore, the critical band regime allows to achieve switching with a minimum energy cost in a broad pulse duration range. However, the core of the critical band is resilient against the thermal effect even thus the boundaries are blurred by the fluctuations (see supplementary material for details). We can conclude that the optimization of the magnetization reversal mechanism depends on two parameters: the *H*_*ip*_*/H*_*k*_ ratio and the damping value *α*. Since these two parameters control the switching time, hereafter we perform an analysis based on the magnetization trajectories on the energy landscape.

It is possible to have an analytical estimation of the characteristic times describing the motion of the magnetization on the surface energy. Therefore, on the energy surface (see Eq. (6) Methods and Fig. 3), one can define isolines as the ones passing by the *Oz* pole ($\theta = 0^\circ$) according to the relation:

4 $$\frac{1}{2}\left( {1 - p} \right) H_{K0} { }\left( {1 - m_{z}^{2} } \right) - H_{ip} m_{x} = 0$$

Using the conservation of the magnetization norm, the magnetization components of any point on this isoline of constant energy are related as $m_{x} = \frac{{1 - m_{z}^{2} }}{r}$ and $m_{y} = \pm \sqrt {1 - m_{z}^{2} - \left( {\frac{{1 - m_{z}^{2} }}{r}} \right)^{2} }$ with $= \frac{{2H_{ip} }}{{\left( {1 - p} \right)H_{K0} }}$. This isoline corresponds to the conservative trajectory of the magnetization described by the precessional term from the LLG (Eq. (7), Methods) (i.e*.* damping $\alpha = 0$). The motion of the magnetization can be decomposed in two intervals (Fig. 3b). First, the magnetization goes from the *Oz* pole to the extreme value reachable by $m_{y}$, during the lapse of time $\tau_{1} \cong \frac{{m_{y}^{max} }}{{\gamma_{0} H_{ip} }}$, approximately $\tau_{1} = \frac{1}{{\gamma_{0} H_{ip} }}\frac{r}{2}$. Second, the magnetization evolution between the extreme $m_{y}^{max}$ and *xOy* plane takes a time $\tau_{2} = \frac{{m_{z} (m_{y}^{max} )}}{{\gamma_{0} H_{ip} m_{y}^{max} { }}}$ which is approximately $\tau_{2} = \frac{{\sqrt {1 - \frac{{r^{2} }}{2}} }}{{\gamma_{0} H_{ip} \frac{r}{2}}}$.

Finally, the duration of motion necessary to reach the *xOy* hard plane is:

5 $$\tau_{1} + \tau_{2} = \frac{1}{{\gamma_{0} H_{ip} }}\frac{r}{2} + \frac{{\sqrt {1 - \frac{{r^{2} }}{2}} }}{{\gamma_{0} H_{ip} \frac{r}{2}}}$$

**Figure 6:**
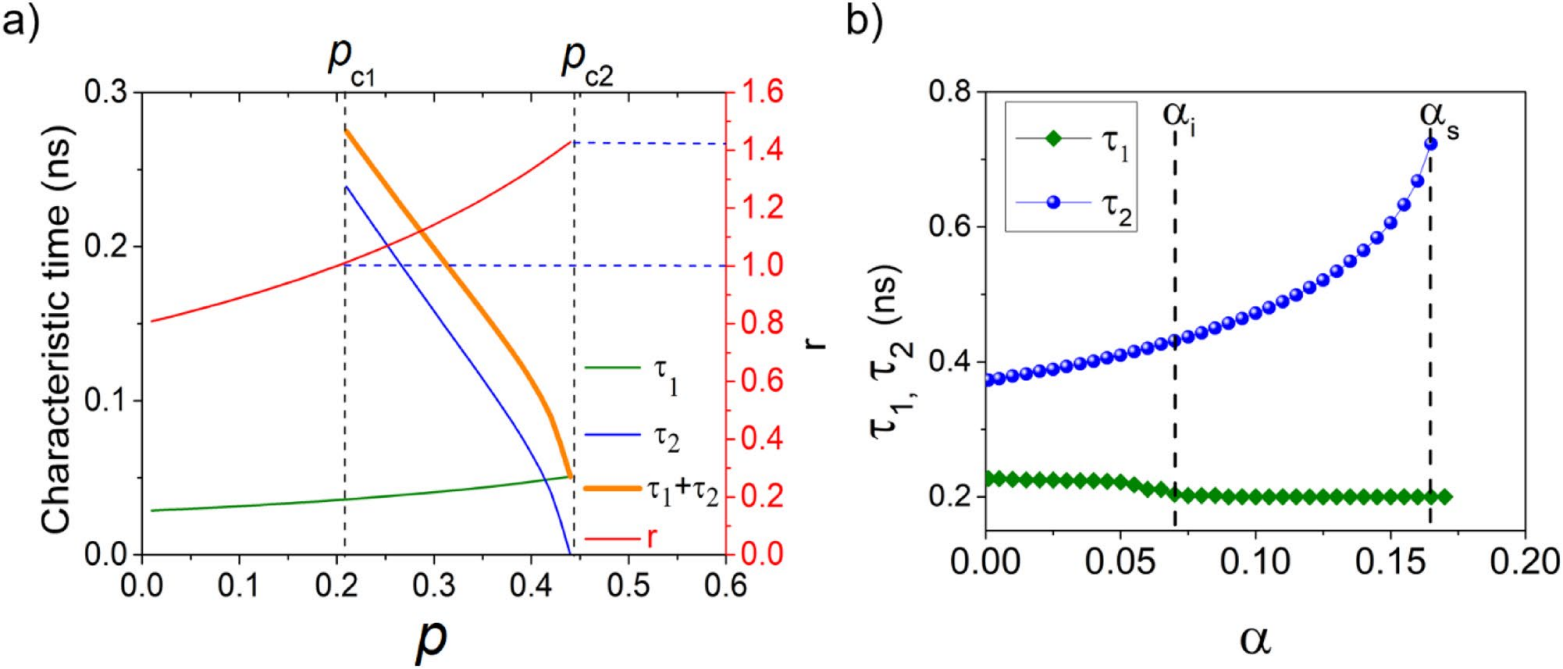
(**a**) Characteristic switching times dependence on *r* and *ξ* for a *H*_*ip*_ = 0.0324 T, from analytical estimation (α = 0); (**b**) Characteristic times and dependence on damping, for a pulse duration of 0.65 ns, *p* = 0.35 and *H*_*ip*_ = 0.0324 T.

Figure 6 shows the control parameters of the magnetization dynamics.

First to be remarked in Fig. 6a is the variation of the characteristic times *τ*_*1*_ and *τ*_*2*_ deduced analytically as functions of the anisotropy modulation, *p*. The sum τ_1_ + τ_2_ can be interpreted as the minimum value of the pulse duration to cross the hard-plan and be able to commute. One can observe that the sum τ_1_ + τ_2_ is relevant and possible only between the critical values *p*_c1_ and *p*_c2_. If *p*_<_ *p*_c1,_ the magnetization trajectory does not reach the hard-plane and τ_2_ is not defined. The value *p*_c1_ coincides with that given by Eq. (2). If *p*_>_ *p*_c2,_ the extreme $m_{y}^{max}$ is lying precisely in the hard-plane and thus the τ_2_ value is vanishing. The characteristic time *τ*_*1*_ is increasing with the modulation *p* while *τ*_*2*_ is decreasing as does also their sum. Regarding the ratio $r$, which enters in the expression of τ_1_ and τ_2_, its values are between 1 and $\sqrt 2$, condition to be fulfilled to have the switching in the regime of critical bands.

In Fig. 4a, we have observed a minimum in the switching time around a modulation *p* = 0.56, for α = 0.01. This minimum shifts to *p* = 0.64 when increasing the damping at α = 0.05. When representing the analytic values of τ1 and τ2 dependences on the damping, we can observe that such a minimum is expected when m_y_ reaches its maximum value at the same time at which m_z_ reaches zero.

Concerning the influence of the damping over the characteristic times τ_1_ and τ_2_, we studied a set of dynamics issued for the following parameters: *p* = 0.35, H_ip_ = 0.0324 T and a pulse duration of 0.65 ns (Fig. 6b). We chose a broad damping interval as it covers large damping values experimentally attainable^37,38^. We observe that τ_1_ decreases slowly with damping up to α = α_i_ = 0.07, from where it stays constant. In contrast, the τ_2_ increases with α, until a threshold α_s_ = 0.165, above which the switching is not possible anymore. The damping range defined by αi and αs values is corresponding to a critical band region, as discussed hereafter.

**Figure 7:**
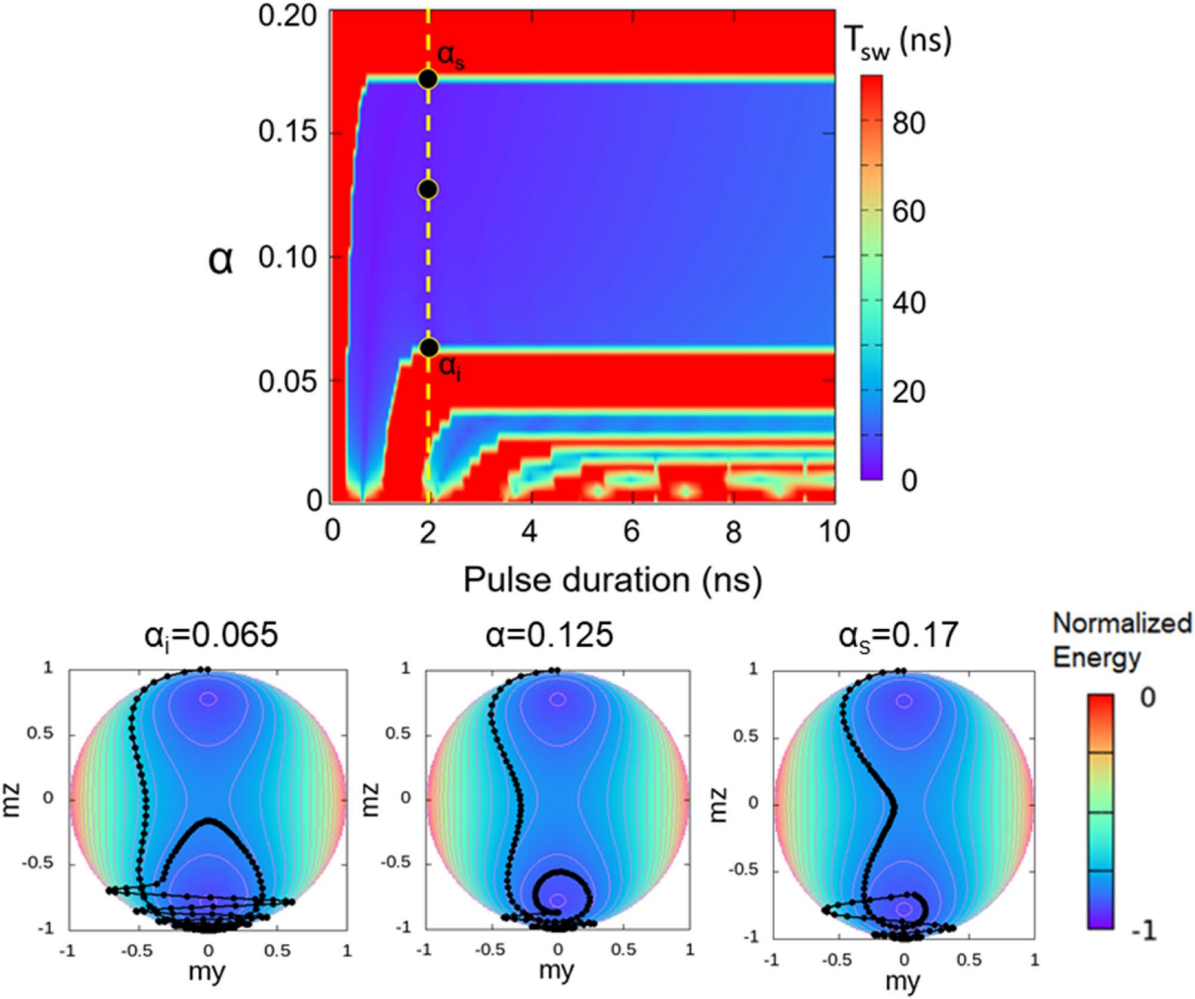
(Top panel) Effect of the damping parameter α on the magnetization switching time. The simulation is performed with *p* = 0.35 and *H*_*ip*_ = 0.0324 T; the switching probability is related with the switching time. (Bottom panel) Magnetization trajectories and normalized energies represented for the damping values α_i_, α and α_s_ represented in the top panel.

Figure 7 summarizes the pathways for magnetization reversal optimization in the VCMA framework. For a given pulse duration, a large damping value favors shorter switching times, as it helps to dissipate energy faster and attenuate the ringing at a higher rate. On the vertical band centered around 0.65 ns, one can appreciate the beneficial effect of damping in terms of switching speed (dark blue color), as the resonant regime identifiable at the bottom of the stripe for a narrow pulse duration range becomes broader. It is obvious that large damping value makes the pulse duration requirements much more flexible in terms of length even for resonant regimes. However, the most interesting aspect in terms of applications remains the existence of critical bands in which the switching probability becomes independent of the pulse duration, corresponding in particular to the large horizontal band, between the α_i_ and α_s_ damping values. Underneath the diagram, three trajectories are shown corresponding to the black circles on the dotted line. This band is the most efficient in terms of switching speed, but new materials or structures with large damping values are necessary. One can notice also that the secondary bands present a similar behavior, leading to the conclusion that for materials with small damping constants as those currently used in applications, it is still possible to achieve such operation regime. As detailed in the supplementary material, the influence of the thermal fluctuations on the horizontal band is to reduce the width of the band and blur the band limits where a stochastic behavior of the magnetization is very likely. However, the core of the band is not affected by the temperature and the concepts discussed for T = 0 K remain valid.

## Conclusions

This work analyses the precessional switching in the VCMA framework in a perpendicularly magnetized nanostructure, emphasizing the idea that there are three parameters which can be adjusted in order to have an energy consumption as small as possible: anisotropy modulation, in-plane magnetic field and damping. We identified the possibility of reaching a regime where the magnetization manipulation can be done independently of the pulse length, because the switching probability is mainly controlled by the ratio between the in-plane field and the modulated anisotropy field, and is maximized for a wider domain by damping. This would be interesting from an application standpoint, as it is easier to work in a wide critical band, where the switching probability is not limited to a small set of parameters and does not depend on an accurately controlled pulse duration. However, working within this critical band, where the deterministic switch is independent on the pulse length, requires additional control of the operating window for the MCA modulation coefficient *p*. Large operating windows could be achieved by tailoring (enhancing) the damping in ferromagnetic heterostructures and/or increasing the value of the in-plane field used for magnetization precession. Another solution for efficient deterministic switching would be to operate the device in a parameter window corresponding to the curved (“elbow” like) region of the bands in the diagrams (see Fig. 2) in which resonant switching also contributes.

Our study is focused on the case where a magnetic in-plane field is applied together with a voltage pulse, in a synchronized manner, but several other pulse configurations can be imagined and explored on the basis of this simple explanation. The electric field manipulation of magnetization renews the interest for purely precessional switching, as the switching condition (the ratio between the in-plane and the anisotropy fields) is actively tuned and choosing materials with a high damping value could increase reliability. We show that there are two leading methods for switching optimization in this framework: one that follows the improvement of the switching time by forcing a faster relaxation in the opposite energy minimum, and one that offers a generous domain for pulse application, with the achievement of similar switching times, thus eliminating this constraint in applications. This combined with the simultaneous cutting of the in-plane magnetic field and voltage pulses, which restores the initial energy barrier between the + *Oz* and  *− Oz* states, can materialize into a new approach for VCMA-based switching technique.

## Methods

The calculations have been performed using a home-made LLG macrospin code developed in C++ and parallelized for CPU computation facilities. In our simulations we have used a free energy of the system, given by:

6 $$E = \left[ { - \frac{1}{2}\mu_{0} H_{K} M_{s} m_{z}^{2} - \mu_{0} M_{s} H_{ip} m_{x} } \right]V$$

with V the sample volume, depends on the time through the magnetization components but also by the time variation of the magnetic field pulse $H_{ip} \left( t \right)$ and that of the amplitude of the anisotropy field $H_{K} \left( t \right)$. The magnetization evolves in time according to the Landau–Lifshitz–Gilbert equation:

7 $$\left( {1 + \alpha^{2} } \right)\frac{{d{\varvec{m}}}}{dt} = - \gamma \left( {{\varvec{m}} \times \mu_{0} {\varvec{H}}_{{{\varvec{eff}}}} } \right) - \alpha \gamma \left[ {{\varvec{m}} \times \left( {{\varvec{m}} \times \mu_{0} {\varvec{H}}_{{{\varvec{eff}}}} } \right)} \right]$$

where ${\varvec{H}}_{{{\varvec{eff}}}} \left( t \right) = \left( {H_{ip} \left( t \right), 0, H_{K} \left( t \right)m_{z} \left( t \right)} \right)$ is the effective field, $\alpha$ is the Gilbert damping parameter, γ is the gyromagnetic ratio and μ_0_ the vacuum permeability. The electric field action on the magneto-crystalline anisotropy (MCA) is accounted by a modulation related to the relative reduction of anisotropy at a given applied electric field value (such as $H_{K} = \left( {1 - p} \right) H_{K0}$ with $0 \le p \le 1$ , $H_{K0}$ being the amplitude of the anisotropy field with no electric field applied. The amplitude modulation can be afterwards rescaled by the precise value of the theoretical VCMA coefficient β, often found in literature under the ξ notation in experimental determinations, as *p* can be translated as a percentage reduction of the effective anisotropy.

## Supplementary Information

Supplementary Information.
