# Supplementary material for: Route towards efficient magnetization reversal driven by voltage control of magnetic anisotropy
Source: Sci Rep. 2021 Apr 22;11:8801. doi: 10.1038/s41598-021-88408-z (PMC8062633; doi:10.1038/s41598-021-88408-z)
Supplement: Supplementary file 1 — Supplementary Information. [file 41598_2021_88408_MOESM1_ESM.docx]

**Supplementary Material**

**Route towards efficient magnetization reversal driven**

**by voltage control of magnetic anisotropy**

Roxana-Alina One^1, 2^ , Hélène Béa^2^, Sever Mican^1^, Marius Joldos^3^ , Pedro Brandão Veiga^2^, Bernard Dieny^2^, Liliana D. Buda-Prejbeanu^2*^, Coriolan Tiusan^1, 3*^

^1^ Faculty of Physics, Babes-Bolyai University, Cluj-Napoca, Cluj, Romania.

^2^ Univ. Grenoble Alpes, CEA, CNRS, G-INP, IRIG-SPINTEC, Grenoble, France.

^3^ Technical University of Cluj-Napoca, Cluj-Napoca, Romania.

*** Corresponding authors:** [coriolan.tiusan@phys.utcluj.ro](mailto:coriolan.tiusan@phys.utcluj.ro), [liliana.buda@cea.fr](mailto:liliana.buda@cea.fr)

This material is meant to be a completion of our work, providing more details about the simulation conditions, limitations and domain of application.

1. **The current induced Rashba field in a ferromagnetic stripe**

The in-plane field *H_ip_* required for the processional reversal may have several origins, ranging from an applied DC field, exchange-bias or Rashba effect. As demonstrated in the literature [1], to generate a net Rashba field a net charge current has to flow through the ferromagnetic field, attainable in a specially designed geometry of a spintronic device (e.g. 3-terminal magnetic tunnel junction in reference [1]). In the absence of a net charge current, from symmetry arguments, the net Rashba field *H_R_* in a ferromagnetic film cancels: the *-k* and the *+k* states are equally populated so that the average $\left\langle k \right\rangle$ would be zero.

To further sustain the choice of using the Rashba field as in-plane field for processional reversal experiments, we performed some theoretical calculations. They provide the experimental conditions in which an in-plane field *H_ip_* =0.0324T (like the one used in our macrospin calculations) could be obtained by a charge net current in a ferromagnetic stripe. We used a theoretical model describing a thin film heterostructure: Au/Fe(5ML)/MgO providing perpendicular magnetic anisotropy. The band structure characteristics (the Fermi wave vector $k_{F}$ and the Rashba coefficient *α_R_* in the Fe film) are extracted from ab-initio *Full Potential Linear Augmented Plane Wave* *FP-LAPW* code *Wien2k* [2] involving a supercell model which describes the experimental multilayer configuration (Fig S3 -top panel).

Beyond the ingredients for the Rashba field estimation, our *ab-inito* calculation provides an anisotropy energy value of 1.37 mJ/m^2^ (calculated as the difference between the total energy corresponding to the in-plane and out-of-plane magnetization configurations). This corresponds to an PMA field *H_K_*=0.08T (dipolar correction $2\pi M_{s}^{2}$ included) that is the one used in our macrospin calculation. The Rashba coefficient $\alpha_{R}$ is extracted from $\alpha_{R}=2E_{0}/k_{0}$, where $E_{0}$is the Rashba splitting at the wave vector $k_{0}$.

| 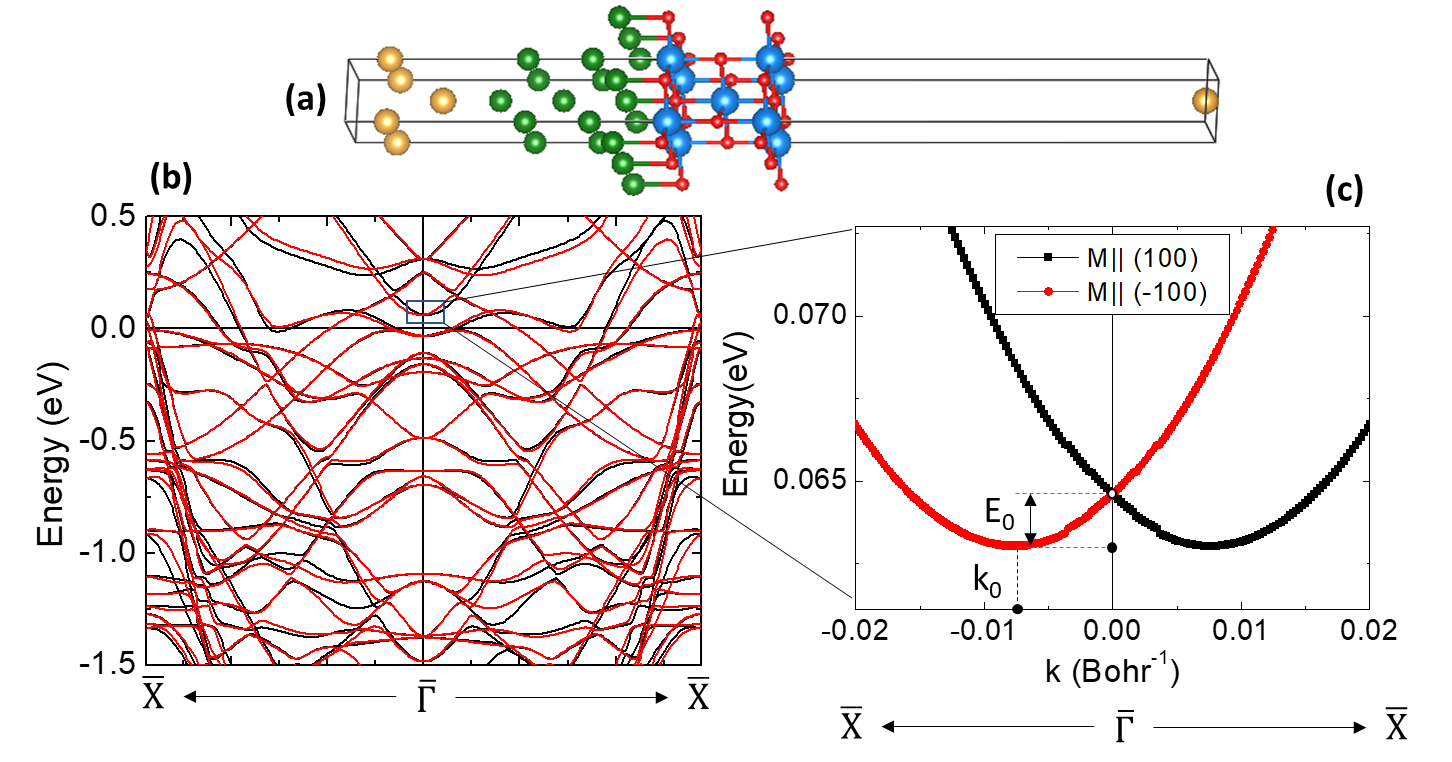 |
| --- |
| **Fig. S1.** (a) Supercell model used for ab-initio calculations in Au/Fe/MgO system. (b) Band-structure of Au/Fe(5ML)/MgO, ML=monolayers when the magnetization is set along the along the $(100)$ -black and $\left( \bar{1}00 \right)$ - red directions. (c) Zoom in the band structure around the $\bar{\Gamma}$ point to calculate the Rashba coefficient. |

The fig. S.1 illustrates the Rashba band splitting of the Au/Fe(5ML)/MgO system, putting the magnetization along the $(100)$ and $\left( \bar{1}00 \right)$ directions. With the calculated $k_{F}=1.0531Å^{-1}$ and $\alpha_{R}=215meVÅ$ one can estimate a Rashba field $H_{R}=\alpha_{R}k_{F}$ that could attain extremely large values (~237meV corresponding to 2370 T). However, for a zero net charge current, *H_R_* cancels out from symmetry reasons: the *–k* and *+k* states are equally populated, and *<k>*=0). In the presence of a charge current, the electron distribution in the *k* space becomes asymmetric leading to $\left\langle k \right\rangle$ ≠0 and *H_R_* ≠0. Theoretical models available in literature [3] [4] quantify the Rashba field associated with the net charge current flow through a ferromagnetic material, as:

$\vec{H}_{R}\approx\frac{\alpha_{R}}{\mu_{B}M_{s}}P\left( \vec{e}_{z}\times\vec{j}_{e} \right)$ (1)

where, *P* is a parameter describing the spin polarization of the conduction electrons in ferromagnet, *M*_s_= 1.714×10^6^ Am^-1^ (the Fe magnetization), $\alpha_{R}=215meVÅ$ , *μ_B_* = 9.274×10^−24^ JT^-1^ is the Bohr magnetron, *j*_e_ = the charge current density (Acm^-2^), $\vec{e}_{z}$= the unit vector of the *z* axis (electric field direction). Considering *P* = 1, corresponding to the Δ_1_ Bloch symmetry electrons in Fe [5] (for other type/symmetry of electrons *P*<1), we get a ratio *H_R_*/*j_e_*~2.15×10^-7^ T cm^2^A^-^1. This leads to a net charge density *j_e_*=1.5041×10^5^ A/cm^2^ that would be required to get a Rashba field *H_R_*=0.0324T, as the one used in our simulations. This field is easily experimentally attainable, e.g. produced by a charge current of *I* =0.15μA flowing in a 1nm thick and 100nm wide ferromagnetic Fe stripe. Similar values can be obtained in other standard PMA heterostructures such as Pt/Co/MgO with similar Rashba coefficient values [6] as our Au/Fe/MgO structure. Therefore, no conceptual limitation is identified, as well as many ”standard” heterostructures *X*/FM/MgO compatible with the MTJ architectures (*X* =Pt, Au, Pd, V, etc., FM=Fe, Co, CoFeB), known to provide perpendicular magnetic anisotropy and VCMA, fulfil the requests. We mention the fact that, this field is smaller than the critical current density required for switching by spin-orbitronic effects, *j*_c_ =3.71×10^5^ A/cm^2^, roughly estimated from 1 by setting the Rashba field to be equal to the perpendicular anisotropy field *H_R_* = *H_K_*).

In order to match with our macrospin simulation framework, in experiments, the Rashba field related to a net charge current *H_ip_=H_R_* has to be synchronized with the voltage pulse responsible on the perpendicular anisotropy E-field control in such a manner that it would restore the effective field along the *Oz* direction after the pulse is cut.

| 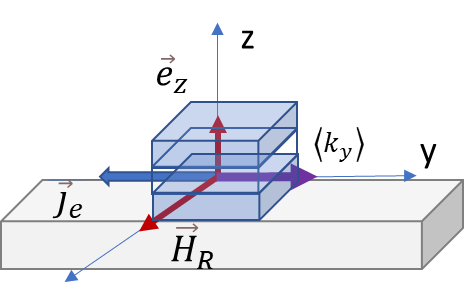 |
| --- |
| **Fig. S2.** Sketch of a 3-terminal MTJ device geometry using the Rashba field generated by planar current in the heavy metal underlayer (in our case in the Au underlayer stripe in the Au/Fe/MgO stack). |

A possible implementation experimental geometry of our concept is illustrated in figure S2, in perfect agreement with the reference [1] of the supplementary ([25] of the main manuscript). Following the eq. (1), the in-plane Rashba field H_R_ can be generated by an in-plane electric current j_e_ perpendicular to both the Rashba field and the electric field directions. To fulfill the planar current request, a current through the heavy metal line under the MTJ device is needed, leading to a 3-terminal geometry of the final device [1]. This current would certainly add a power consumption penalty during the writing stage. However, the penalty could be partially moderated by the fact that, for the writing stage, the planar current pulse is only applied is a very short pulse fully synchronized with the voltage pulse that controls the magnetic anisotropy (as in our simulations). Moreover, having in view the extremely short pulse time length, one can also eliminate the concerns regarding a decrease of the thermal stability factor.

1. **Thermal fluctuations influence on the critical band width**

The main purpose of our study was to describe the magnetization dynamics in the VCMA framework, by phenomenologically decomposing its motion and mapping its behavior within a set of material properties and experimental constraints. In order to develop such an analysis, we considered that approaching it first without the thermal noise blur, will allow us to observe clearly the behavior in the critical points. However, after we established what parameters control the dynamics and in what manner, we also wanted to explore the situation in which the thermal fluctuations are present in the macrospin’s environment. Therefore, a significant part of our work has been performed again, but taking into account a temperature of 300 K, as we are interested to verify if the features discovered in the switching diagram at 0 K will be available at ambient temperature. If figure S3(a), we took as a starting point the diagram issued for a damping constant of 0.05 and an in-plane field of 0.0324 T, which can be observed in the main text as Fig. 2(d). In this figure, we focused our attention on the area enclosed in the rectangle and by doing so, we have defined p_s_ and p_i_ as the edge-values of the critical modulation, for which at 0 K, the pulse-independent switching is allowed. The thermal fluctuations have been introduced in our model as a thermal field proportional to the temperature and damping, with a zero mean-average [7][8]:

$H_{th}=\sqrt{\frac{2\alpha k_{B}T_{sim}}{\gamma_{0}\mu_{0}M_{s}V_{sim}\Delta t}}$ (2)

Where α -damping parameter, $k_{B}$ – Boltzmann constant, $T_{sim}$ -simulation temperature, $\gamma_{0}$ – gyromagnetic ratio, $\mu_{0}$ -magnetic permeability, $M_{s}$- saturation magnetization, $V_{sim}$ – the simulation volume and $\Delta t$- the simulation time step, This led us to obtain a noisy diagram as in Fig. S3(b). As one can see, by comparison, the features from 0 K can be also observed under thermal fluctuations. Hence, we also considered it useful to present this result through a histogram of the critical band profile. We conclude that the thermal fluctuations narrow the critical band, for critical modulations around p_i_ and p_s_, thus narrowing down the domain of the working regime.

A similar analysis was done for Fig. 7 from the main text (here displayed as Fig. S3(a)), where for a well-established critical modulation p=0.35 and a H_ip_=0.0324 T, the damping constant was varied between 0 and 0.2.


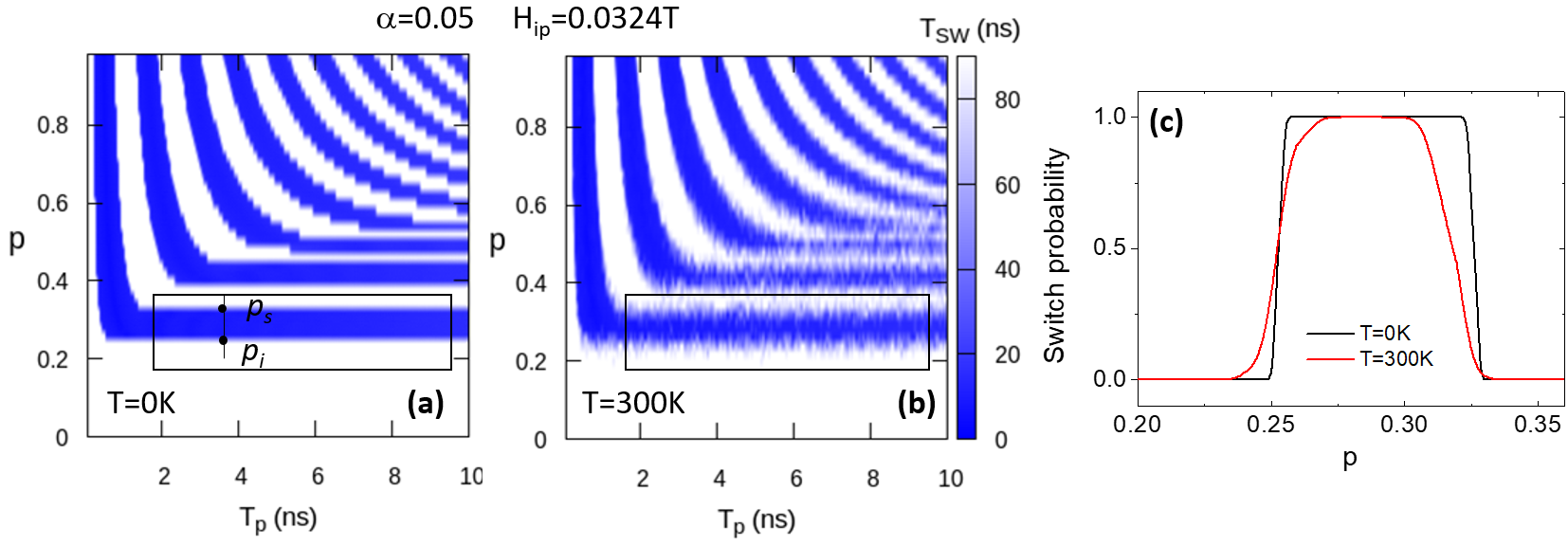


**Fig. S3.** Switching diagrams for *α*=0.05, *H_ip_*=0.0324 T at (a) 0 K and (b) 300 K. (c) Superposed critical band profile at 0 K and 300 K.

Fig. S4(b) shows that the features from T=0 K are conserved at 300 K, but the same effect of the thermal fluctuations has been observed in the peripheral regions of the pulse-length independent band. The working regime is narrowing and from the band profile histogram (in Fig. S4(c)), we can observe that this time it is narrowing in an asymmetric way. This can be explained through the fact that the thermal field is directly proportional to the damping constant, which means that a larger value of the damping will boost the effect of the thermal fluctuations, as it was also indicated by [9].


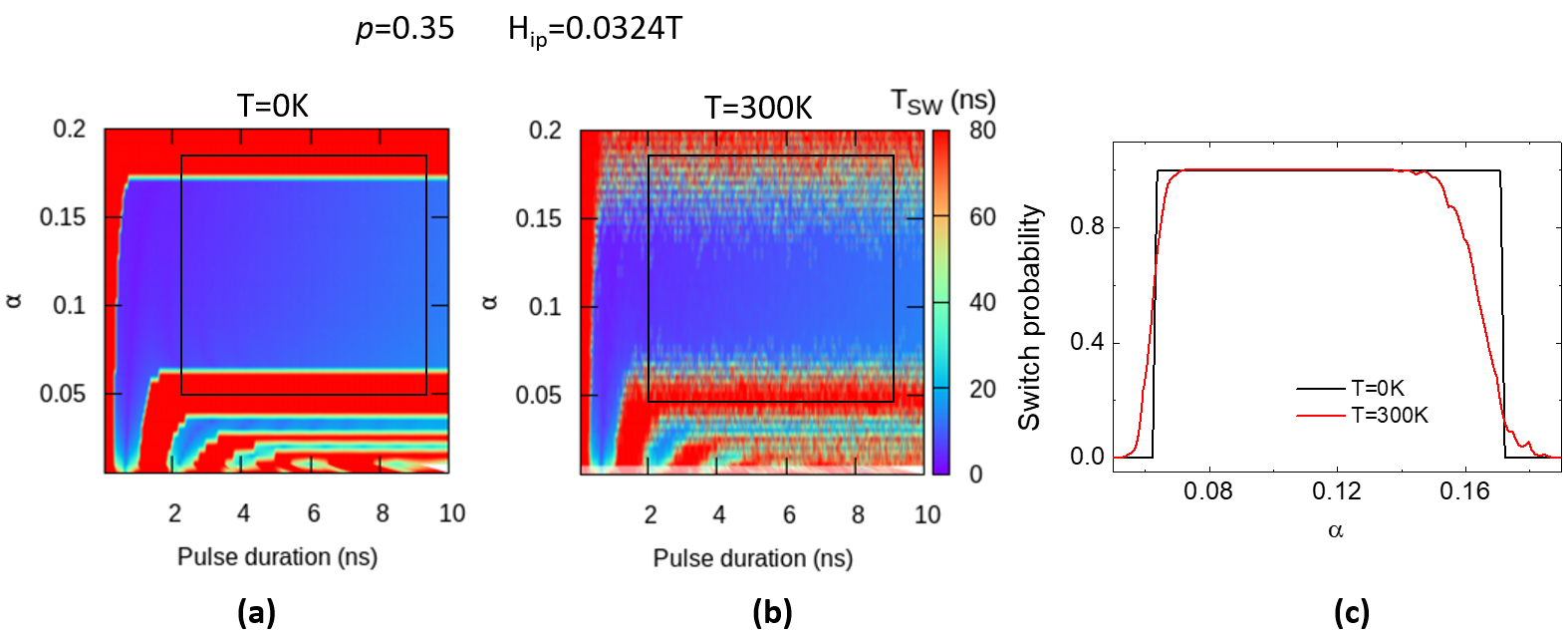


**Fig. S4.** Switching diagrams for *p*=0.35 and *H_ip_*=0.0324 T at (a) 0 K and (b) 300 K. (c) Superposed critical band profiles at 0 K and 300 K illustrating the thermal induced asymmetric narrowing and the noise on band boundaries.

To sum up the conclusions from both Fig. S3 and Fig. S4, increasing the temperature will rather alter the peripheral regions of the critical bands, but their core is resilient against the thermal fluctuations. One possibility to solve this issue would be to apply a larger in-plane field, so that the core of the band would be close to p=0. By doing so, the inferior border of the critical line will be out of discussion, making it easier to have a precise control on the modulation and with extremely small electric field. Moreover, beyond the fluctuations induced by the thermal field (H_fl_), the temperature may affect the ferromagnetic materials parameters such as the saturation magnetization M_s_ and the PMA which both depend on temperature [7] [10]. Therefore, when the macrospin calculations must be compared with experiments, the experimental variation of M_s_ and K_s_ with the temperature should be carefully considered.

**Supplementary references**

[1] Deng, J., Fong, X. & Liang, G. Electric-field-induced three-terminal pMTJ switching in the absence of an external magnetic field. *Appl. Phys. Lett.* **112**, 252405 (2018).

[2 Blaha, P, Schwarz, K., Tran, F., Laskowski, R., Madsen, G. K. H., and Marks, L.D., WIEN2k: An APW+lo program for calculating the properties of solids, J. Chem. Phys. **152**, 074101 (2020).

[3] Manchon, A. & Zhang, S., Theory of nonequilibrium intrinsic spin torque in a single nanomagnet, Phys. Rev. B **78**, 212405 (2008).

[4] Miron, I.M.M., Gaudin, G., Auffret, S., Rodmacq, B., Schuhl, A., Pizzini, S., Vogel, J., & Gambardella, P., Current-driven spin torque induced by the Rashba effect in a ferromagnetic metal layer. Nat Mater. **9**, 230–234 (2010).

[5] Butler, W. H., Zhang, X.-G., Schulthess, T.C. & MacLaren, J. M. Spin-dependent tunneling conductance of Fe|MgO|Fe sandwiches. Phys. Rev. B **63**, 054416 (2001).

[6] Yang, H., Boulle, O., Cros, V., Fert, A. & Chshiev, M. Controlling Dzyaloshinskii-Moriya Interaction via Chirality Dependent Atomic-Layer Stacking, Insulator Capping and Electric Field. Sci. Rep. **8**, 12356 (2018).

[7] N. Strelkov, N., Chavent, A., Timopheev, A., Sousa, R. C., Prejbeanu, I. L., Buda-Prejbeanu, L. D., Dieny, B., Impact of Joule heating on the stability phase diagrams of perpendicular magnetic tunnel junctions, Phys. Rev. B **98**, 214410 (2018).

[8] Hahn, M.B., Temperature in micromagnetism: cell size and scaling effects of the stochastic Landau–Lifshitz equation, *J. Phys. Commun.* **3**, 075009 (2019).

[9] Shiota, Y., Nozaki, T. , Tamaru, S., Yakushiji, K., Kubota, H., Fukushima, A. , Yuasa, S., Suzuki, Y., Evaluation of write error rate for voltage-driven dynamic magnetization switching in magnetic tunnel junctions with perpendicular magnetization. *Appl. Phys. Express* **9**, 013001 (2016).

[10] Gabor, M.S, Petrisor, T., Pop, O., Colis, S., Tiusan, C., Temperature dependence of the perpendicular magnetic anisotropy in Ta/Co2FeAl/MgO structures probed by Anomalous Hall Effect, *J. Magn. Magn. Mater.* **392**, 79-82 (2015).
